# Outcomes of Endovascular Therapy in Acute Basilar Artery Occlusion With Severe Symptoms

**DOI:** 10.1001/jamanetworkopen.2021.39550

**Published:** 2021-12-16

**Authors:** Weilin Kong, Junjie Yuan, Jiacheng Huang, Jiaxing Song, Chenhao Zhao, Hongfei Sang, Weidong Luo, Dongjing Xie, Fei Gao, Huagang Li, Jun Luo, Shudong Liu, Dongzhang Xue, Yinquan Yu, Fengli Li, Zhongming Qiu, Wenjie Zi, Qingwu Yang

**Affiliations:** 1 Department of Neurology, Xinqiao Hospital and the Second Affiliated Hospital, Army Medical University (Third Military Medical University), Chongqing, China; 2 Department of Neurology, Zhongnan Hospital, Wuhan University, Wuhan, China; 3 Department of Neurology, 404th hospital of Mianyang, Mianyang, China; 4 Department of Neurology, Yongchuan Hospital of Chongqing Medical University, Chongqing Key Laboratory, Chongqing, China; 5 Department of Neurology, 902nd Hospital of the People’s Liberation Army, Bengbu, China; 6 Department of Neurology, Bazhong Hospital of Traditional Chinese Medicine, Bazhong, China

## Abstract

**Importance:**

Endovascular therapy (EVT) has been reported to be safe and effective in improving clinical outcomes among patients with acute basilar artery occlusion (ABAO). The benefits associated with EVT remain uncertain for patients with ABAO with severe symptoms (ie, National Institutes of Health Stroke Scale [NIHSS] score ≥ 21).

**Objectives:**

To assess the outcomes associated with EVT and identify factors associated with outcomes among patients with ABAO and severe symptoms.

**Design, Setting, and Participants:**

This prospective, nationwide cohort study was conducted using data from January 2014 to May 2019 in China from the Endovascular Treatment for Acute Basilar Artery Occlusion Study Registry (BASILAR). Included patients had ABAO and underwent EVT or standard medical treatment (SMT) alone in routine clinical practice. Patients were dichotomized into severe symptoms (ie, NIHSS score ≥ 21) and minor to moderate symptoms (NIHSS score < 21) groups. Patients were followed up for 90 days. Data were analyzed from December 2020 through June 2021.

**Exposures:**

EVT with SMT vs SMT alone.

**Main Outcomes and Measures:**

The primary outcome was improvement in modified Rankin Scale (mRS) score at 90 days, defined as a decrease by 1 grade in mRS score. Secondary outcomes included favorable functional outcome (ie, mRS score, 0-3) and mortality.

**Results:**

Among 542 patients with ABAO and severe symptoms (median [IQR] age, 65 [57-74] years; 147 [27.1%] women), 431 patients (79.5%) received EVT and 111 patients (20.5%) received SMT. Compared with SMT, EVT was associated with increased odds of improved mRS score (adjusted common odds ratio [OR], 3.44 [95% CI, 2.05-5.78]; *P* < .001), with increased odds of a favorable functional outcome (ie, mRS score, 0-3; adjusted OR, 4.52 [95% CI, 1.64-12.43]; *P* = .004) and decreased odds of mortality (adjusted OR, 0.27 [95% CI, 0.15-0.50]; *P* < .001). Among patients receiving EVT, baseline NIHSS score was associated with decreased odds of a favorable functional outcome (adjusted OR per 1-point increase in score, 0.90 [95% CI, 0.85-0.95]; *P* < .001) and increased odds of mortality (adjusted OR per 1-point increase in score, 1.13 [95% CI, 1.07-1.19]; *P* < .001), while posterior circulation Acute Stroke Prognosis Early Computed Tomography Score (pc-ASPECTS) was associated with increased odds of a favorable functional outcome (adjusted OR per 1-point increase in score, 1.71 [95% CI, 1.41-2.07]; *P* < .001) and decreased odds of mortality (adjusted OR per 1-point increase in score, 0.74 [95% CI, 0.64-0.85]; *P* < .001). Different occlusion sites, compared with distal basilar artery, were associated with decreased odds of favorable functional outcome (eg, middle basilar artery: adjusted OR vs distal basilar artery, 0.36 [95% CI, 0.17-0.80]; *P* = .01).

**Conclusions and Relevance:**

This study found that EVT was associated with increased odds of functional favorable outcomes among patients with ABAO and severe symptoms. Baseline NIHSS score, pc-ASPECTS, and occlusion site were independent factors associated with clinical outcomes.

## Key Points

**Question:**

Is endovascular therapy associated with improved outcomes among patients with acute basilar artery occlusion and severe symptoms?

**Findings:**

In this cohort study of 542 patients with acute basilar artery occlusion and severe symptoms, endovascular therapy was associated with increased odds of improvement in modified Rankin Scale scores at 90 days compared with standard medical treatment alone.

**Meaning:**

These findings suggest that endovascular therapy should be considered an effective strategy for treatment of acute basilar artery occlusion with severe symptoms among selected patients.

## Teaser

This cohort study investigates the association of endovascular therapy with modified Rankin Scale score compared with standard medical treatment among patients with acute basilar artery occlusion and severe symptoms.

## Introduction

Although acute basilar artery occlusion (ABAO) is an infrequent ischemic stroke, accounting for a small proportion (approximately 1%) of all ischemic strokes and 5% to 10% of strokes resulting from large vessel occlusion,^1,2^ it usually represents a disastrous disease associated with high rates of disability and mortality.^1,2,3^ Given that standard medical treatment (SMT) alone is associated with insufficient recanalization rates and poor outcomes among patients with ABAO, endovascular therapy (EVT) has recently been regarded as a promising therapeutic approach for ABAO.^2,4^ Since 2006, several studies^5,6,7,8^ of the efficacy and safety associated with EVT in ABAO have found results suggesting that EVT may be associated with safe and effective improvements in clinical outcomes and decreased mortality among patients with ABAO. However, the Basilar Artery International Cooperation Study (BASICS) and Basilar Artery Occlusion Chinese Endovascular Trial (BEST) study did not find sufficient evidence of a difference in favorable outcomes among patients receiving EVT compared with those receiving SMT,^2,9,10^ but the latest results of the BASICS randomized clinical trial may not exclude a substantial benefit of EVT.^10^ Encouragingly, our cohort study (Endovascular Treatment for Acute Basilar Artery Occlusion Study [BASILAR]) found that EVT was associated with better functional outcomes and decreased mortality within 24 hours among patients with ABAO.^11^ In a hopeful finding, the latest study, by Nappini et al from 2021,^12^ found that patients with ischemic stroke due to BAO treated with bridging and direct EVT had decreased mortality and better functional outcome in ordinal analysis at 3 months compared with other studies. These complicated findings suggest that the efficacy of EVT may be associated with clinical severity in ABAO.

A 2021 study^13^ found that EVT was associated with safer and more effective treatment among patients with mild BAO strokes (ie, National Institutes of Health Stroke Scale [NIHSS] score ≤ 6) than among patients with more severe disease. Specifically, patients with minor to moderate stroke had an increased rate of favorable outcomes (ie, modified Rankin Scale [mRS] score, 0-2) and a decreased rate of periprocedural complications and mortality after mechanical thrombectomy (MT) compared with patients with severe symptoms.^14^ Although MT has a high rate of recanalization, a significant proportion (approximately 32%) of patients with mild ABAO had a poor long-term clinical outcome.^13^ Interestingly, Wang et al^6^ found that EVT was associated with effective restoration of cerebral blood flow and decreased incidence of complications among patients with ABAO and severe symptoms. This finding suggests that clinical severity at admission may be associated with neurological outcomes after EVT. Whether EVT is associated with improved outcomes among patients with ABAO and severe symptoms (ie, NIHSS score ≥ 21) remains unknown, and the factors associated with functional outcome after EVT remain to be elucidated.

In this study, we sought to compare clinical outcomes after EVT vs SMT among patients with ABAO and severe symptoms. We additionally sought to investigate prognostic factors associated with clinical outcomes.

## Methods

### Study Design, Setting, and Data Collection

This cohort study used the registry data set of BASILAR, a multicenter, observational study including 829 consecutive patients confirmed to have ABAO from January 2014 to May 2019 in 47 senior stroke centers across 15 provinces in China. The study followed the Strengthening the Reporting of Observational Studies in Epidemiology (STROBE) reporting guideline for cohort studies. Ethics committees of the local institutional review boards of each center approved the study protocol. We obtained written informed consent from patients or their legal authorized representatives according to the Declaration of Helsinki.

### Participants

We included 829 consecutive study participants from the BASILAR registry^11^; all participants centers were required to enter all consecutive patients to avoid selection bias in the study. Eligible patients met the following criteria: (1) were aged at least 18 years; (2) presented with acute, symptomatic angiographically confirmed BAO within 24 hours of estimated occlusion time or if not known at the last time the patient was observed to have no symptoms before the onset of stroke symptoms; and (3) had undergone intravenous thrombolysis within the therapeutic time window. The choice of treatment was at the discretion of the treating physician of the investigation center. Stroke severity of ABAO was dichotomized into severe symptoms (ie, NIHSS score ≥ 21) and minor to moderate symptoms (ie, NIHSS score < 21) groups using NIHSS score from the BASILAR study.^2,15^ We excluded 287 patients with minor to moderate symptoms and 542 patients with severe symptoms on admission (eFigure 1 in the Supplement). Participants were divided into an EVT group (ie, those receiving SMT with MT, stenting, thrombus aspiration, balloon angioplasty, or a combination of these approaches) and SMT group (ie, those receiving SMT alone, including antiplatelet and anticoagulation treatment, intravenous thrombolysis, or a combination of these therapies). The exact strategies of therapy were left to the judgement of the local neurointerventionist.

### Variables and Outcomes

Correlative neuroimaging data were analyzed by a neuroimaging core laboratory, whose members (F.L., Z.Q., W.Z.) were independent, experienced neuroradiologists who did not know the clinical information. When there was controversy about a patient’s data, the final evaluation was reached by comprehensively considering the input of 2 experienced vascular neurologists (F.L. and Z.Q.) and 1 neuroradiologist (W.Z.). Detailed methods of the BASILAR Registry have been reported previously.^11^ The baseline posterior circulation Acute Stroke Prognosis Early Computed Tomography Score (pc-ASPECTS) was graded according to a previous description.^11,16^ The posterior circulation collateral score (PC-CS) represented the collateral circulation status based on the presence of potential collateral pathways on computed tomography angiography,^17^ and the basilar artery on tomography angiography (BATMAN) score was assessed as the posterior circulation collateral status.^18^

The primary outcome was mRS score at 90 days, with functional improvement defined as a decrease in mRS score by 1 grade. The mRS score is a 7-level categorical scale with scores ranging from 0 (no symptoms) to 6 (death). Secondary outcomes included functional outcomes that were excellent (ie, mRS score, 0-1), showing functional independence (ie, mRS score, 0-2), or favorable (ie, mRS score, 0-3); mortality at 90 days; and symptomatic intracranial hemorrhage based on the Heidelberg bleeding classification. Successful recanalization was defined as modified thrombolysis in cerebral infarction (mTICI) score of 2b to 3. This scale ranges from 0 (no reperfusion) to 3 (complete recanalization) based on angiographic data.^19^

### Statistical Analysis

Categorical and binary variables were compared using χ^2^ or Fisher exact tests, while continuous variables were compared using Student *t* test (ie, mean comparison) for variables with normal distributions and Mann-Whitney U test for variables without normal distributions.

For baseline characteristics and outcomes, normally distributed continuous variables were presented by their mean and SD, nonnormally distributed continuous variables and ordinal variables were presented as median and IQR, and categorical variables were presented as absolute numbers and percentages. We compared binary outcome (ie, favorable outcome vs poor outcome) for EVT vs SMT groups and performed shift analysis on mRS score using multivariable binary logistic regression and ordinal logistic regression analysis. For regression analyses with favorable outcomes and mortality, we adjusted for the following prognostic factors: age, sex, baseline systolic blood pressure (SBP), smoking history, atrial fibrillation status, baseline NIHSS score, baseline PC-CS, baseline pc-ASPECTS, baseline BATMAN score, stroke etiology, occlusion site, intravenous thrombolysis (IVT) status, and onset treatment time.

We also generated benefit curves by analyzing baseline NIHSS score to investigate the association between NIHSS score and favorable functional outcomes at 90 days using multivariable binary logistic regression. We evaluated heterogeneity of the association of EVT with functional outcomes across dichotomized NIHSS score subgroups by producing an interaction term. We plotted the probabilities of favorable functional outcome, and we presented adjusted odds ratios (ORs) with 95% CIs. The 3-dimensional distribution surfaces diagram represents the probabilities of the estimated outcomes, which were found using SigmaPlot software version 14 (SPSS) and with models assessed using the R^2^ correlation metric. For propensity score matching analysis, we performed 1:2 matching based on the nearest-neighbor matching algorithm with a caliper width of 0.2 × the propensity score, with age, baseline NIHSS score, baseline pc-ASPECTS, stroke etiology, occlusion site, and IVT status included in the analysis as covariables. We adjusted for these covariables in multifactor analysis (eMethods 1 in the Supplement).

Statistical analyses were performed using SPSS statistical software version 26 (IBM) and Stata statistical software version 16 (StataCorp). The level of statistical significance was considered at 2-tailed *P* < .05. We excluded patients with missing essential data from our analysis, so we did not impute for missing data (eMethods 2 in the Supplement). Data were analyzed from December 2020 through June 2021.

## Results

### Baseline Characteristics

Among 542 patients with ABAO and severe symptoms, the median (IQR) age and baseline NIHSS score were 65 (57-74) years and 30 (27-35), respectively; there were 147 (27.1%) women and 395 (72.9%) men. Among all patients, 431 patients (79.5%) received EVT and 111 patients (20.5%) received SMT. After 1:2 propensity score matching, baseline characteristics achieved favorable balance in the EVT vs SMT groups (median [IQR] age, 64 [56-74] years vs 66 [59-75] years; *P* = .21; 155 [77.9%] men vs 71 [67.6] men; *P* = .05). A comparison of baseline characteristics among patients with EVT vs SMT is provided in Table 1.

**Table 1.** Baseline Patient Characteristics

Characteristic	Unmatched patients, No. (%)			*P* value	Propensity score–matched patients, No. (%)			*P* value
	Total (N = 542)	EVT (n = 431)	SMT (n = 111)		Total (N = 304)	EVT (n = 199)	SMT (n = 105)	
Age, median (IQR), y	65 (57-74)	64 (57-74)	66 (59-75)	.15	65 (57-74)	64 (56-74)	66 (59-75)	.21
Sex								
Women	147 (27.1)	111 (25.8)	36 (32.4)	.16	78 (25.7)	44 (22.1)	34 (32.4)	.05
Men	395 (72.9)	320 (74.2)	75 (67.6)		226 (74.3)	155 (77.9)	71 (67.6)	
NIHSS score, median (IQR)	30 (27-35)	30 (27-35)	32 (28-35)	.17	32 (28-35)	32 (28-35)	32 (29-35)	>.99
pc-ASPECTS, median (IQR)^a^	8 (6-9)	8 (6-9)	7 (6-8)	.001	7 (6-8)	8 (6-8)	7 (6-8)	.20
PC-CS, median (IQR)	4 (3-6)	4 (3-5)	4 (3.5-6)	.05	4 (3-6)	4 (3-6)	4 (4-6)	.26
BATMAN score, median (IQR)	4 (2-5)	3 (2-5)	4 (2-6)	.008	4 (2-5)	3 (2-5)	4 (2-6)	.01
Body temperature, median (IQR)^b^	36.7 (36.5-36.9)	36.7 (36.5-36.8)	36.7 (36.5-37.0)	.02	36.6 (36.5-36.9)	36.6 (36.5-36.8)	36.7 (36.5-37.0)	.06
SBP, median (IQR)^c^, mm Hg	151 (135-170)	150 (134-167)	160 (144-171)	.001	151 (136-166)	147 (133-161)	160 (145-172)	<.001
DBP level, median (IQR)^c^, mm Hg	85 (77-97)	85 (75-96)	88 (80-100)	.05	85 (78-96)	84 (75-96)	87 (80-99)	.09
Vascular risk factor								
Smoking, current or past	179 (33.0)	160 (37.1)	19 (17.1)	<.001	94 (30.9)	75 (37.7)	19 (18.1)	<.001
Hypertension	388 (71.6)	308 (71.5)	80 (72.1)	.90	213 (70.1)	137 (68.8)	76 (72.4)	.52
Hyperlipidemia	173 (31.9)	133 (30.9)	40 (36.0)	.30	103 (33.9)	65 (32.7)	38 (36.2)	.54
Diabetes	128 (23.6)	103 (23.9)	25 (22.5)	.76	69 (22.7)	47 (23.6)	22 (21.0)	.60
Drinking, current or past	123 (22.7)	102 (23.7)	21 (18.9)	.29	64 (21.1)	44 (22.1)	20 (19.0)	.53
Medical history								
Atrial fibrillation	120 (22.1)	104 (24.1)	16 (14.4)	.03	59 (19.4)	43 (21.6)	16 (15.2)	.18
Coronary artery disease	103 (19.0)	87 (20.1)	16 (14.4)	.17	55 (18.1)	39 (19.6)	16 (15.2)	.35
Heart failure^d^	19 (3.5)	17 (3.9)	2 (1.8)	.39	9 (3.0)	7 (3.5)	2 (1.9)	.72
Prodrome	252 (46.5)	197 (45.7)	55 (49.5)	.47	141 (46.4)	88 (44.2)	53 (50.5)	.30
Cerebral infarction	134 (24.7)	101 (23.4)	33 (29.7)	.17	78 (25.7)	49 (24.6)	29 (27.6)	.57
Intracerebral hemorrhage^d^	10 (1.8)	8 (1.9)	2 (1.8)	.97	4 (1.3)	2 (1.0)	2 (1.9)	.61
Chronic bronchitis^d^	8 (1.5)	8 (1.9)	0	.37	5 (1.6)	5 (2.5)	0 (0)	.17
Stroke etiology								
LAA	339 (62.5)	268 (62.2)	71 (64.0)	.004	196 (64.5)	129 (64.8)	67 (63.8)	.25
CE	143 (26.4)	123 (28.5)	20 (18.0)		68 (22.4)	48 (24.1)	20 (19.0)	
Other causes	60 (11.1)	40 (9.3)	20 (18.0)		40 (13.2)	22 (11.1)	18 (17.1)	
Occlusion site								
Distal BA	188 (34.7)	155 (36.0)	33 (29.7)	.04	90 (29.6)	60 (30.2)	30 (28.6)	.93
Middle BA	175 (32.3)	117 (27.1)	58 (52.3)		158 (52.0)	102 (51.3)	56 (53.3)	
Proximal BA	76 (14.0)	70 (16.2)	6 (5.4)		15 (4.9)	9 (4.5)	6 (5.7)	
VA-V4	103 (19.0)	89 (20.7)	14 (12.6)		41 (13.5)	28 (14.1)	13 (12.4)	
IVT	131 (24.2)	93 (21.8)	38 (34.2)	.009	88 (28.9)	57 (28.6)	31 (29.5)	.87
OTT, median (IQR), min	311 (141-402)	308 (144-401)	244 (140-451)	.75	235 (132-393)	233 (132-373)	244 (138-458)	.34
Recanalization	350 (64.6)	341 (79.1)	9 (8.1)	<.001	162 (53.3)	154 (77.4)	8 (7.6)	<.001

Abbreviations: BA, basilar artery; BATMAN, basilar artery on tomography angiography; CE, cardioembolism; DBP, diastolic blood pressure; EVT, endovascular therapy; IVT, intravenous thrombolysis; LAA, large artery atherosclerosis; NIHSS, National Institutes of Health Stroke Scale; OTT, onset treatment time; pc-ASPECTS, posterior circulation Acute Stroke Prognosis Early Computed Tomography Score; PC-CS, posterior circulation collateral score; SBP, systolic blood pressure; SMT, standard medical treatment; VA-V4, vertebral artery V4 segment.

^a^ Data were missing for 4 patients in the EVT group and 2 patients in the SMT group.

^b^ Data were missing for 6 patients in the EVT group and 1 patient in the SMT group.

^c^ Data were missing for 2 patients in the EVT group.

^d^ *P* values were calculated using Fisher exact tests.

### Outcomes of EVT vs SMT Stratified by Severity

**Figure 1.**
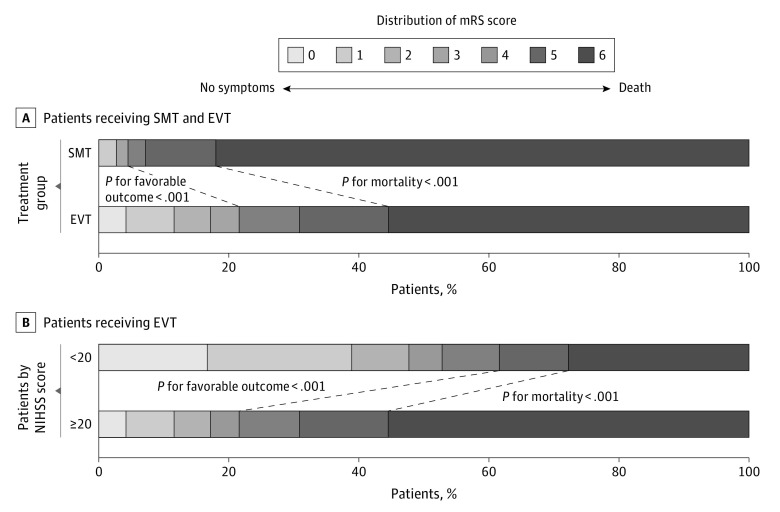
Modified Rankin Scale (mRS) Scores at 90 Days The distributions of the mRS score for favorable functional outcomes and mortality among patients in the standard medical treatment (SMT) and endovascular therapy (EVT) groups are presented among all patients and those receiving EVT. NIHSS indicates National Institutes of Health Stroke Scale.

EVT was associated with increased rates of favorable functional outcome (93 patients [21.6%] vs 5 patients [4.5%]; OR, 5.83 [95% CI, 2.31-14.72]; *P* < .001) and decreased rates of mortality (239 patients [55.5%] vs 91 patients [82.0%]; OR, 0.27 [95% CI, 0.16-0.46]; *P* < .001) at 90 days compared with the SMT group in unadjusted analysis (Figure 1A; eTable 1 in the Supplement). EVT was also associated with better outcomes compared with SMS when that was defined as functional improvement as measured by mRS score (common OR, 3.44 [95% CI, 2.05-5.78]; *P* < .001), with increased odds of a favorable functional outcome (ie, mRS score, 0-3; adjusted OR, 4.52 [95% CI, 1.64-12.43]; *P* = .004) and decreased odds of mortality (adjusted OR, 0.27 [95% CI, 0.15-0.50]; *P* < .001). A statistically significant difference was observed for functional outcome with EVT vs SMS (excellent outcome: 50 patients [11.6%] vs 3 patients [2.7%]; OR, 3.20 [95% CI, 0.89-11.57]; *P* = .08; showing functional independence: 74 patients [17.2%] vs 3 patients; OR, 5.55 [95% CI, 1.59-19.38]; *P* = .007) in adjusted analysis. The rates of symptomatic intracranial hemorrhage were increased with EVT compared with SMT (40 patients [9.5%] vs 1 patient [0.9%]; OR, 10.26 [95% CI, 1.34-78.62]; *P* = .03) (eTable 1 in the Supplement). After 1:1 propensity score matching analysis, the results were consistent with multivariable regression logistic analysis with adjustment for covariates and are presented in eTable 1 in the Supplement.

**Figure 2.**
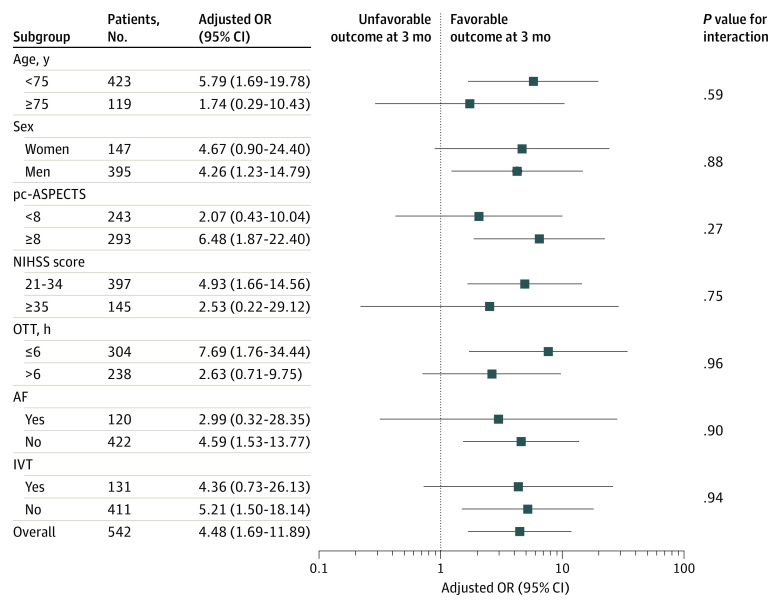
Subgroup Analyses of Clinical Outcomes The forest plot shows the differences in odds ratios (ORs) for favorable outcomes at 90 days in the prespecified subgroups. Adjusted variables include age, sex, smoking history, atrial fibrillation (AF) status, stroke etiology, occlusion site, intravenous thrombolysis (IVT) status, posterior circulation Acute Stroke Prognosis Early Computed Tomography Score (pc-ASPECTS), and onset treatment time (OTT). NIHSS indicates National Institutes of Health Stroke Scale.

**Figure 3.**
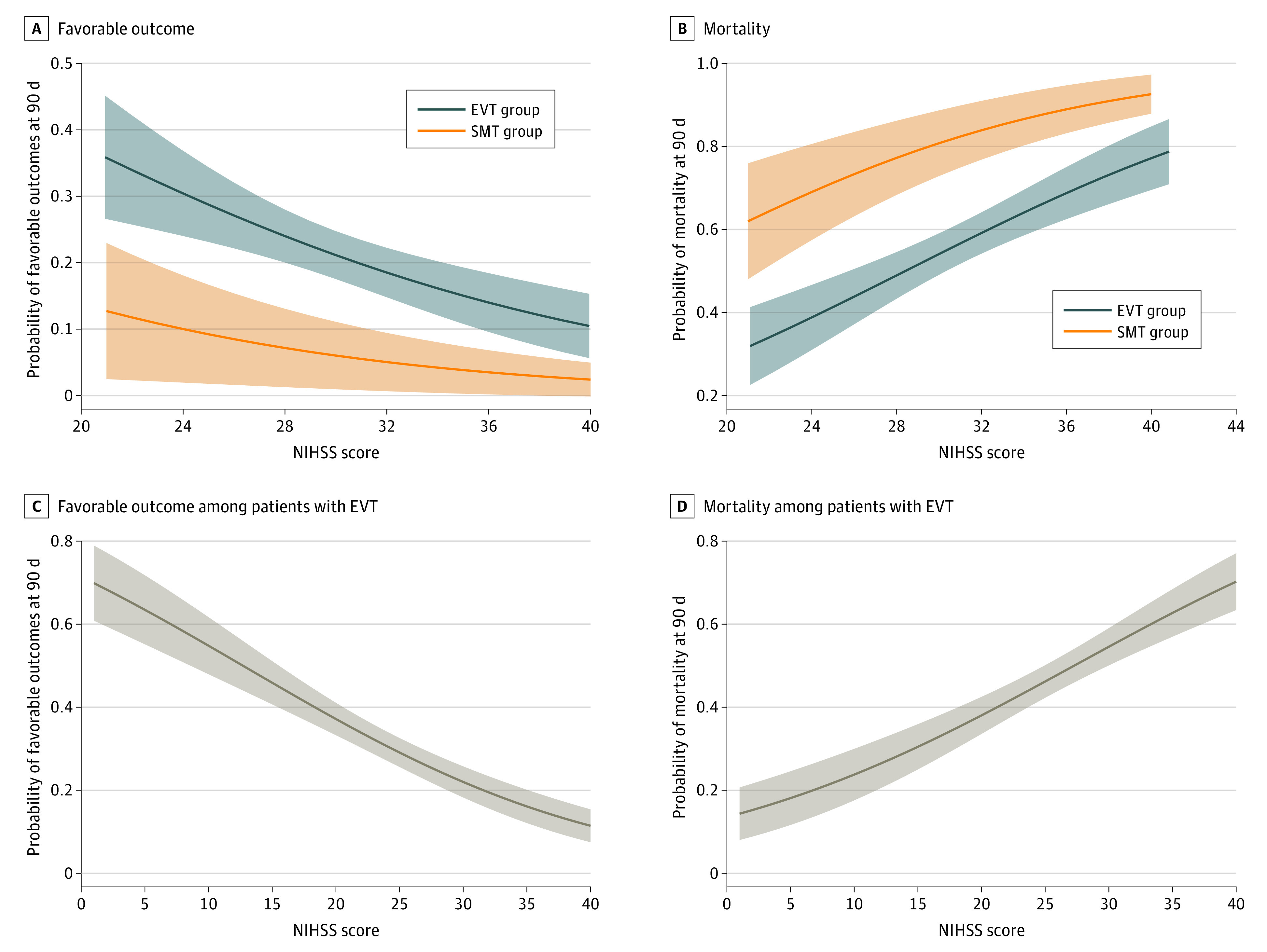
Association of Baseline National Institutes of Health Stroke Scale (NIHSS) With Probability of Clinical Outcomes The estimated probabilities of a favorable outcome and mortality by NIHSS score among all patients with severe ABAO are presented in A and B. Among patients with severe ABAO, the endovascular therapy (EVT) group had increased estimated probability of favorable functional outcome and decreased estimated probability of mortality than the standard medical treatment (SMT) group. The estimated probabilities of favorable functional outcome and mortality by NIHSS score among patients receiving EVT are presented in C and D. Among all patients with EVT, increased NIHSS score was associated with decreased estimated probabilities of favorable functional outcomes and increased estimated probabilities of mortality. Solid lines indicate estimated probabilities of outcomes; shaded areas, 95% CIs.

Among patients in both EVT and SMS groups, baseline NIHSS score was associated with decreased estimated probability of favorable functional outcome (adjusted OR per 1-point increase in score, 0.90 [95% CI, 0.85-0.95]; *P* < .001) and increased estimated probability of mortality (adjusted OR per 1-point increase in score, 1.12 [95% CI, 1.08-1.17]; *P* < .001). Baseline NIHSS score was associated with a decrease in the odds of favorable outcome of 12.0% (adjusted OR per 1-point increase in NIHSS score, 0.88 [95% CI, 0.83-0.94]; *P* < .001) in the EVT group and 4.0% in the SMT group (adjusted OR per 1-point increase in NIHSS score, 0.96 [95% CI, 0.77-1.19]; *P* = .77), which was not a statistically significant change. Baseline NIHSS score was associated with an increase in the odds of mortality of 13.0% in the EVT group (adjusted OR per 1-point increase in NIHSS score, 1.13 [95% CI, 1.07-1.19]; *P* < .001); there was a 15.0% increase in odds of mortality in the SMT group (adjusted OR per 1-point increase in NIHSS score, 1.15 [95%CI, 0.99-1.33]; *P* = .14), but this increase was not statistically significant (Figure 2A and B). In the analysis adjusted for prognostic factors, EVT was associated with increased odds of a functional outcome (adjusted OR, 3.80 [95% CI, 1.36-10.58]; *P* = .01) and decreased odds of mortality (adjusted OR, 0.29 [95% CI, 0.16-0.54]; *P* < .001) compared with SMT (eTable 2 in the Supplement). There was an interaction association of baseline NIHSS score and onset to treatment time, age, glucose level, and SBP with decreased probabilities of favorable functional outcome per 1-point increase in NIHSS score (eFigure 2 in the Supplement). In the results of the subgroup analyses, there was no significant heterogeneity across any prespecified subgroup (Figure 3).

### Factors Associated With Outcome for EVT With Severe Symptoms

Restricting the analysis to just the EVT group, patients were dichotomized by functional outcome (ie, favorable vs poor) (eTable 3 in the Supplement) and mortality (ie, living vs dead) to investigate factors associated with outcome after EVT by univariate and logistic regression analyses. In the analysis adjusted for prognostic factors, baseline NIHSS score (OR per 1-point increase in NIHSS score, 0.90 [95% CI, 0.85-0.95]; *P* < .001), baseline pc-ASPECTS (adjusted OR per 1-point increase in pc-ASPECTS, 1.71 [95%CI, 1.41-2.07]; *P* < .001), occlusion site (eg, middle basilar artery: adjusted OR vs distal basilar artery, 0.36 [95% CI, 0.17-0.80]; *P* = .01), and successful recanalization (adjusted OR vs no successful recanalization, 4.96 [95%CI, 1.79-13.74]; *P* = .002) were factors associated with functional outcome. Additionally, baseline SBP (adjusted OR per 1 mm Hg increase in SBP, 1.01 [95% CI, 1.00-1.02]; P = .04), baseline NIHSS score (adjusted OR per 1-point increase in score, 1.13 [95%CI, 1.07-1.19]; *P* < .001), baseline pc-ASPECTS (adjusted OR per 1-point increase in score, 0.74 [95%CI, 0.64-0.85]; *P* < .001), and successful recanalization (adjusted OR vs no successful recanalization, 0.17 [95%CI, 0.09-0.34]; *P* < .001) were factors associated with mortality (Table 2).

**Table 2.** Multivariate Analysis of Estimators of Outcome After Endovascular Therapy

Variable	Unadjusted OR (95% CI)	*P* value	Adjusted OR (95% CI)	*P* value
**Favorable outcome**				
Age^a^	0.99 (0.97-1.01)	.34	1.00 (0.96-1.01)	.35
Body temperature^b^	0.40 (0.23-0.68)	.001	0.44 (0.22-0.86)	.02
Baseline NIHSS score^c^	0.90 (0.85-0.94)	<.001	0.90 (0.85-0.95)	<.001
Baseline PC-CS^c^	1.26 (1.10-1.44)	.001	1.03 (0.81-1.30)	.84
Baseline pc-ASPECTS^c^	1.67 (1.41-1.98)	<.001	1.71 (1.41-2.07)	<.001
Baseline BATMAN score^c^	1.26 (1.11-1.43)	<.001	1.20 (0.96-1.51)	.11
Occlusion site^d^				
Distal BA	1 [Reference]	NA	1 [Reference]	NA
Middle BA	0.39 (0.22-0.72)	.002	0.36 (0.17-0.80)	.01
Proximal BA	0.52 (0.26-1.04)	.06	0.60 (0.23-1.53)	.28
VA-V4	0.44 (0.22-0.87)	.02	0.45 (0.18-1.10)	.08
IVT	1.00 (0.57-1.74)	.99	0.89 (0.46-1.75)	.74
Recanalization	5.91 (2.32-15.05)	<.001	4.96 (1.79-13.74)	.002
**Mortality**				
Age^a^	1.02 (1.00-1.03)	.07	1.02 (1.00-1.04)	.12
Baseline SBP^e^	1.01 (1.00-1.01)	.15	1.01 (1.00-1.02)	.04
Body temperature^b^	1.70 (1.20-2.41)	.003	1.43 (0.95-2.17)	.09
Baseline NIHSS score^c^	1.13 (1.08-1.18)	<.001	1.13 (1.07-1.19)	<.001
Baseline PC-CS^c^	0.85 (0.76-0.95)	.003	0.97 (0.80-1.19)	.77
Baseline pc-ASPECTS^c^	0.73 (0.65-0.83)	<.001	0.74 (0.64-0.85)	<.001
Baseline BATMAN score^c^	0.85 (076-0.94)	.002	0.91 (0.76-1.09)	.30
Recanalization	0.17 (0.09-0.31)	<.001	0.17 (0.09-0.34)	<.001

Abbreviations: BA, basilar artery; BATMAN, basilar artery on tomography angiography; IVT, intravenous thrombolysis; NA, not applicable; NIHSS, National Institutes of Health Stroke Scale; OR, odds ratio; pc-ASPECTS, posterior circulation Acute Stroke Program Early Computed Tomography Score; PC-CS, posterior circulation collateral score; SBP, systolic blood pressure; VA-V4, vertebral artery V4 segment.

^a^ OR is per 1-year increase in age.

^b^ OR is per 0.1-degree Celsius increase in body temperature.

^c^ OP is per 1-point increase in score.

^d^ Distal BA was taken as reference.

^e^ OR is per 1 mm Hg increase.

Baseline NIHSS score was associated with decreased probabilities of favorable functional outcome with EVT. The estimated marginal effects of favorable outcome probabilities on onset to puncture time decreased with increasing baseline NIHSS score and were not statistically significant when NIHSS score was greater than 35 in EVT group (eFigure 3A in the Supplement). The marginal effects of probabilities of favorable outcome on puncture to recanalization time decreased with increasing baseline NIHSS score in the EVT group, and the estimated marginal effect on puncture to recanalization time was not statistically significant when the NIHSS score was 35 or more (eFigure 3B in the Supplement). The association of factors with prognostic outcomes had a steeper benefit slope among patients with increased NIHSS scores (eFigure 4 in the Supplement).

### Association of NIHSS Score With Outcomes in EVT Group

Among 647 patients treated with EVT in the BASILAR study, 431 patients had severe symptoms and 216 patients had minor to moderate symptoms. A comparison of the characteristics among patients with severe symptoms vs patients with minor symptoms (ie, NIHSS score < 10) to moderate symptoms (ie, NIHSS score, 10-20) symptoms is provided in eTable 4 in the Supplement.

In univariate analysis, patients with severe symptoms who received EVT had increased mortality (239 patients [55.5%] vs 60 patients [27.8%]; *P* < .001) and comparable rates of favorable functional outcome (93 patients [21.6%] vs 114 patients [52.8%]; *P* < .001) compared with patients with minor to moderate symptoms (Figure 1B). The estimated probabilities of favorable functional outcome decreased per 1-point increment in baseline NIHSS score in the EVT group, and the estimated probabilities of mortality increased per 1-point increment in baseline NIHSS score (Figure 2C and D). When adjusted for dichotomized NIHSS score and other prognostic factors, increased baseline NIHSS score (ie, NIHSS score ≥ 21) remained a factor associated with decreased odds of favorable outcome and increased odds of mortality (eTable 5 in the Supplement).

## Discussion

This prospective multicentric cohort study investigated real-world clinical experiences to evaluate the outcomes associated with EVT among patients with ABAO and severe symptoms. Our study found that EVT was associated with favorable outcome and better functional outcome among patients with severe ABAO, with a more than 5-fold increase in the likelihood of achieving favorable functional outcome at 90 days. Moreover, EVT was associated with safe treatment of severe ABAO, with a decreased rate of mortality. Additionally, we found that baseline NIHSS score, baseline pc-ASPECTS, occlusion site, and successful recanalization were independent factors associated with favorable functional outcome among patients with ABAO who underwent EVT.

To our knowledge, studies to date have found that posterior circulation stroke was associated with more severe symptoms among patients with very severe stroke (ie, NIHSS score > 25) and that ABAO was associated with impaired consciousness and longer treatment delays.^20^ Although thrombectomy was associated with increased rates of recanalization in mild BAO strokes compared with severe strokes, a substantial proportion of patients (32%) had an inferior long-term clinical outcomes.^13^ Recently, a study^21^ focusing on ABAO with severe symptoms reported that EVT was associated with increased rates of recanalization; however, outcomes have failed to meet expectations among patients with severe posterior circulation steno-occlusive disease. Similarly, intra-arterial MT combined with thrombolysis or stent placement was found to be associated with effective restoration of blood flow and preservation of life, with decreased rates of complications among patients with severe ABAO.^6^ In our study, EVT was associated with effective treatment of patients with severe ABAO, with an increased proportion of patients with a favorable outcome (93 patients [21.6%] vs 5 patients [4.5%]) and decreased mortality (239 patients [55.5%] vs 91 patients [82.0%]) at 90 days compared with SMT. Importantly, the benefit associated with EVT decreased as NIHSS score increased, and there was no statistically significant benefit associated with EVT when NIHSS score was 35 or more. These findings suggest that EVT should be more rigorous among patients with very severe symptoms (ie, NIHSS score ≥ 35). This finding is consistent with previously reported outcomes among patients with ABAO who underwent EVT.^6,9,22,23^ The complicated functional outcomes among ABAO patients who underwent EVT may be associated with stroke severity and multiple prognostic factors.^20,24^

Although a 2012 study^25^ described factors associated with clinical outcomes after EVT among patients with ABAO, few studies have focused on modern thrombectomy among patients with severe ABAO. The previously identified factors independently associated with a good outcome after EVT in posterior circulation stroke included decreased baseline NIHSS score, shorter time to recanalization, increased pc-ASPECTS, shorter time to therapy, absence of prodromal minor stroke, better collateral status, and absence of hyperlipidemia.^13,25^ Patients with ABAO and mild to moderate stroke had better posterior circulation collateral and better outcomes after MT than patients with severe stroke in a 2021 study.^14^ Moreover, younger age, decreased NIHSS score, lack of diabetes, and lack of cerebral hemorrhage were associated with better outcomes in a 2018 study.^23^ Few studies have evaluated independent factors associated with clinical outcome after stent-retriever thrombectomy among patients with severe ABAO.^26^ In our study, decreased NIHSS score, increased pc-ASPECTS, no smoking history, decreased body temperature, occlusion site, recanalization status, and stroke etiology were associated with favorable outcome among patients with severe ABAO. Consistent with a 2019 study,^27^ middle basilar artery site occlusion was an important factor associated with worse prognosis. The middle basilar artery angle is associated with flow bends and hemodynamic flow turbulence and increases in the proportion of patients with atherosclerosis; it was found to be the main factor associated with poor clinical outcomes in middle basilar artery occlusion,^28^ because EVT would be likely to harm the perforators in patients with middle basilar artery occlusion atherosclerotic lesions.^1,27^

A previous study^29^ suggested that NIHSS score and pc-ASPECTS were independent estimators of long-term functional outcome among patients with ABAO treated with EVT. In our study, high baseline NIHSS score and low pc-ASPECTS were associated with mortality among patients with ABAO and severe symptoms. NIHSS score, pc-ASPECTS, SBP, body temperature, and successful recanalization were factors associated with mortality. Additionally, in the setting of recanalization, we found that age, baseline NIHSS score, pc-ASPECTS, and intravenous thrombolysis were also independent factors associated with mortality after EVT among patients with ABAO, which was consistent with the results of Gory et al.^30^ However, while successful recanalization is an indicator of EVT, recanalization was not found to be a prognostic factor associated with clinical outcome.^26,31,32^ Furthermore, our study’s findings suggest that NIHSS score, pc-ASPECTS, body temperature, and SBP are independent factors associated with mortality after EVT among patients with ABAO.

### Limitations

This study has several limitations. Our results must be interpreted within the context of the study design. First, this was an observational study, and the associations of EVT and SMT alone with clinical outcomes were determined in a nonrandomized fashion. Therefore, there may be inevitable selection biases in the treatment of patients. Among patients with severe symptoms, the percentage who underwent EVT was significantly higher than those who received SMT alone. Second, other factors, such as collateral status, core and penumbra mismatch, and prodromal status, were not included in our analysis and would need to be adjusted for in further analysis aiming to estimate the benefit associated with EVT. Additionally, the baseline NIHSS scores of ABAO were high among all patients in our study and would not accurately reflect the critical location or volume of the lesion. Therefore, even among patients with the same NIHSS score, it is difficult to accurately estimate the outcome.

## Conclusions

We found an association of EVT with favorable functional outcome among patients with ABAO. Baseline NIHSS score, baseline pc-ASPECTS, body temperature, baseline SBP, occlusion site, and successful recanalization were identified as independent prognostic factors in ABAO treated with EVT.

## Notes

Supplement. **eMethods 1.** Propensity Score Matching Analysis **eMethods 2.** Missing or Incomplete Data **eTable 1.** Clinical Outcomes of Patients with Severe Acute Basilar Artery Occlusion by Treatment **eTable 2.** Multivariate Analysis of Estimators of Outcomes **eTable 3.** Comparison of Patients With Favorable vs Poor Outcomes **eTable 4.** Baseline Characteristics of Patients with Endovascular Therapy **eTable 5.** Multivariate Analysis of Estimators of Outcomes After Endovascular Therapy **eFigure 1.** Flowchart **eFigure 2.** Association of Baseline National Institutes of Health Stroke Scale Score With Probability of Favorable Outcome **eFigure 3.** Estimated Marginal Effects of Favorable Outcome Probabilities on Onset to Puncture (OTP) Time and Puncture to Recanalization (PTR) Time **eFigure 4.** Association of Baseline National Institutes of Health Stroke Scale Score With Probability of Clinical Outcomes With Endovascular Therapy
