# Supplementary material for: Outcomes of Endovascular Therapy in Acute Basilar Artery Occlusion With Severe Symptoms
Source: JAMA Netw Open. 2021 Dec 16;4(12):e2139550. doi: 10.1001/jamanetworkopen.2021.39550 (PMC8678675; doi:10.1001/jamanetworkopen.2021.39550)
Supplement: Supplement. — eMethods 1. Propensity Score Matching Analysis eMethods 2. Missing or Incomplete Data eTable 1. Clinical Outcomes of Patients with Severe Acute Basilar Artery Occlusion by Treatment eTable 2. Multivariate Analysis of Estimators of Outcomes eTable 3. Comparison of Patients With Favorable vs Poor Outcomes eTable 4. Baseline Characteristics of Patients with Endovascular Therapy eTable 5. Multivariate Analysis of Estimators of Outcomes After Endovascular Therapy eFigure 1. Flowchart eFigure 2. Association of Baseline National Institutes of Health Stroke Scale Score With Probability of Favorable Outcome eFigure 3. Estimated Marginal Effects of Favorable Outcome Probabilities on Onset to Puncture (OTP) Time and Puncture to Recanalization (PTR) Time eFigure 4. Association of Baseline National Institutes of Health Stroke Scale Score With Probability of Clinical Outcomes With Endovascular Therapy [file jamanetwopen-e2139550-s001.pdf]

Supplemental Online Content

Kong W, Yuan J, Huang J, et al. Outcomes of endovascular therapy in acute basilar artery occlusion with severe symptoms. *JAMA Netw Open*. 2021;4(12):e2139550. doi:10.1001/jamanetworkopen.2021.39550

eMethods 1. Propensity Score Matching Analysis
eMethods 2. Missing or Incomplete Data
eTable 1. Clinical Outcomes of Patients with Severe Acute Basilar Artery Occlusion by Treatment
eTable 2. Multivariate Analysis of Estimators of Outcomes
eTable 3. Comparison of Patients With Favorable vs Poor Outcomes
eTable 4. Baseline Characteristics of Patients with Endovascular Therapy
eTable 5. Multivariate Analysis of Estimators of Outcomes After Endovascular Therapy
eFigure 1. Flowchart
eFigure 2. Association of Baseline National Institutes of Health Stroke Scale Score With Probability of Favorable Outcome
eFigure 3. Estimated Marginal Effects of Favorable Outcome Probabilities on Onset to Puncture (OTP) Time and Puncture to Recanalization (PTR) Time
eFigure 4. Association of Baseline National Institutes of Health Stroke Scale Score With Probability of Clinical Outcomes With Endovascular Therapy

This supplemental material has been provided by the authors to give readers additional information about their work.

© 2021 Kong W et al. *JAMA Network Open.*

eMethods 1. Propensity Score Matching Analysis

We performed a 1:2 propensity score matching based on the nearest-neighbor matching algorithm with a caliper width of 0.2 of the propensity score with age, baseline NIHSS, baseline posterior circulation-Acute Stroke Prognosis Early CT Score (pc-ASPECTS), etiology, occlusion site, and IVT. Propensity score matching analysis were performed using the IBM SPSS Statistics 26 version (IBM Corp, Armonk, NY, USA). Six patients were excluded because of lack of baseline pc-ASPECTS score. The variables, parameter settings and results are listed as follows:

```
PSMATCHING3
  /VARS
    TREAT = group
    COVS = baseline NIHSS baseline ASPECTS Occlusion Site IVT
    ADDLCOVS = age TOAST
  /MATCHIT
    MATCH=NEAREST
    EST =LOGIT
    DISCARD = NONE
    MORDER = LARGEST
    RATIO = 2
    CALIPER = .2
  /OPTIONS DETAILEDBAL DETAILEDBAL_CLUSTER
  /PLOT HISTBAL DOTPLOT INDBAL RESOLUTION = 300
  /OUTPUT PAIRED MATCHED_CASES_ONLY.
```

Sample Sizes

Subsamples	All		Matched		Unmatched		Discarded	
	Control	Treated	Control	Treated	Control	Treated	Control	Treated
(all cases)	431	111	199	105	232	6	0	0

© 2021 Kong W et al. *JAMA Network Open.*

Relative multivariate imbalance L1 (Iacus, King, & Porro, 2010)		
	Before matching	After matching
(all cases)	.970	.952

Summary of unbalanced covariates (|d| > .25)
No covariate exhibits a large imbalance (|d| > .25).

R Graph

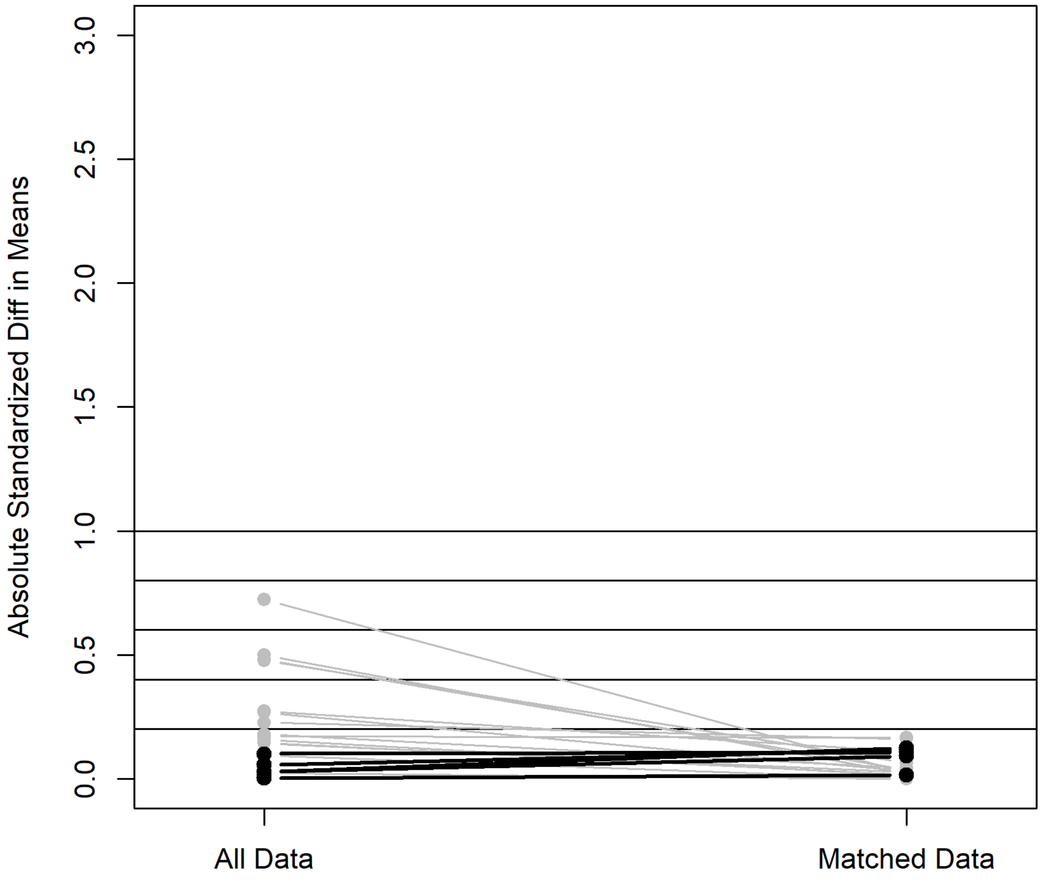

© 2021 Kong W et al. *JAMA Network Open.*

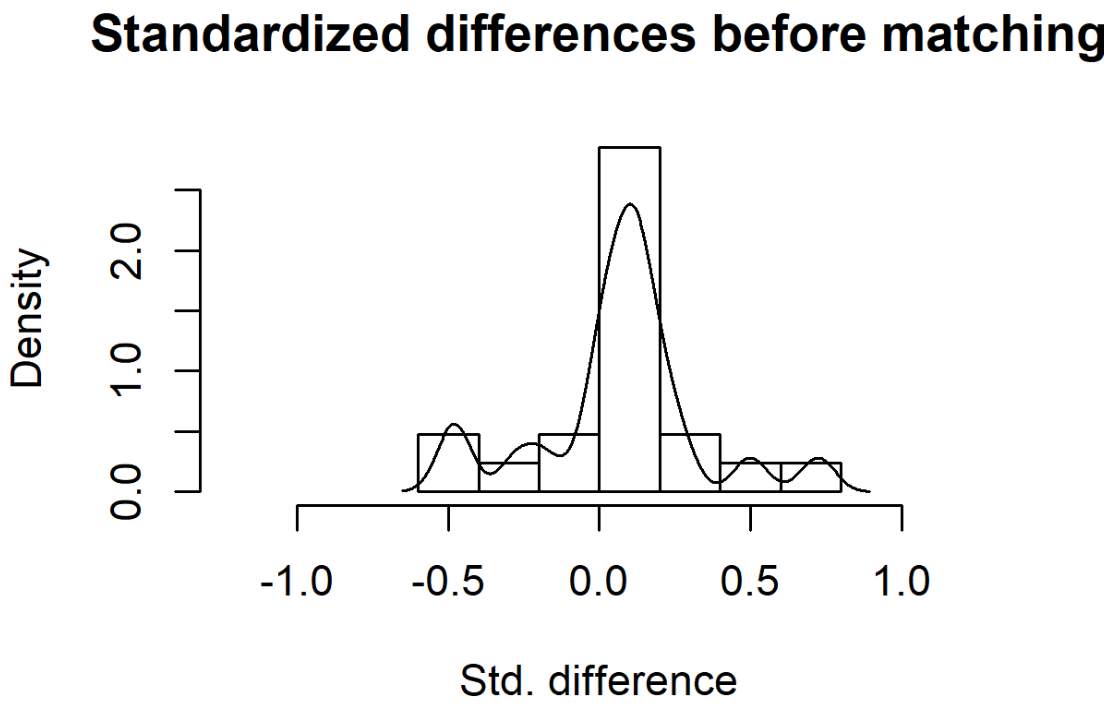

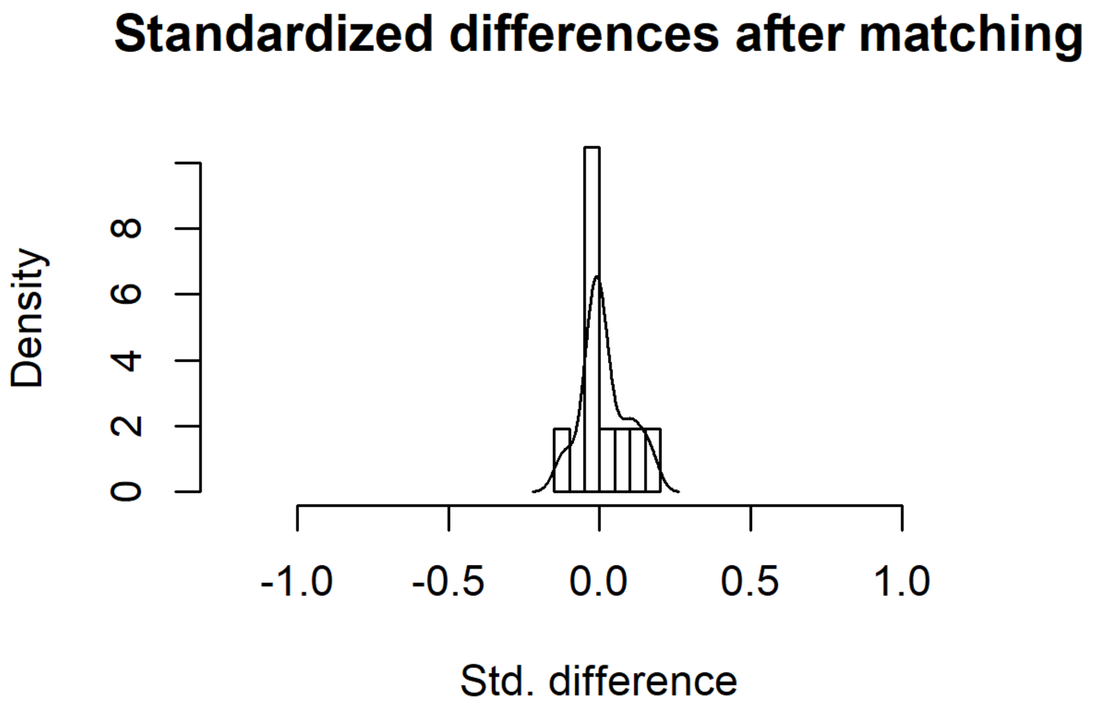

© 2021 Kong W et al. *JAMA Network Open.*

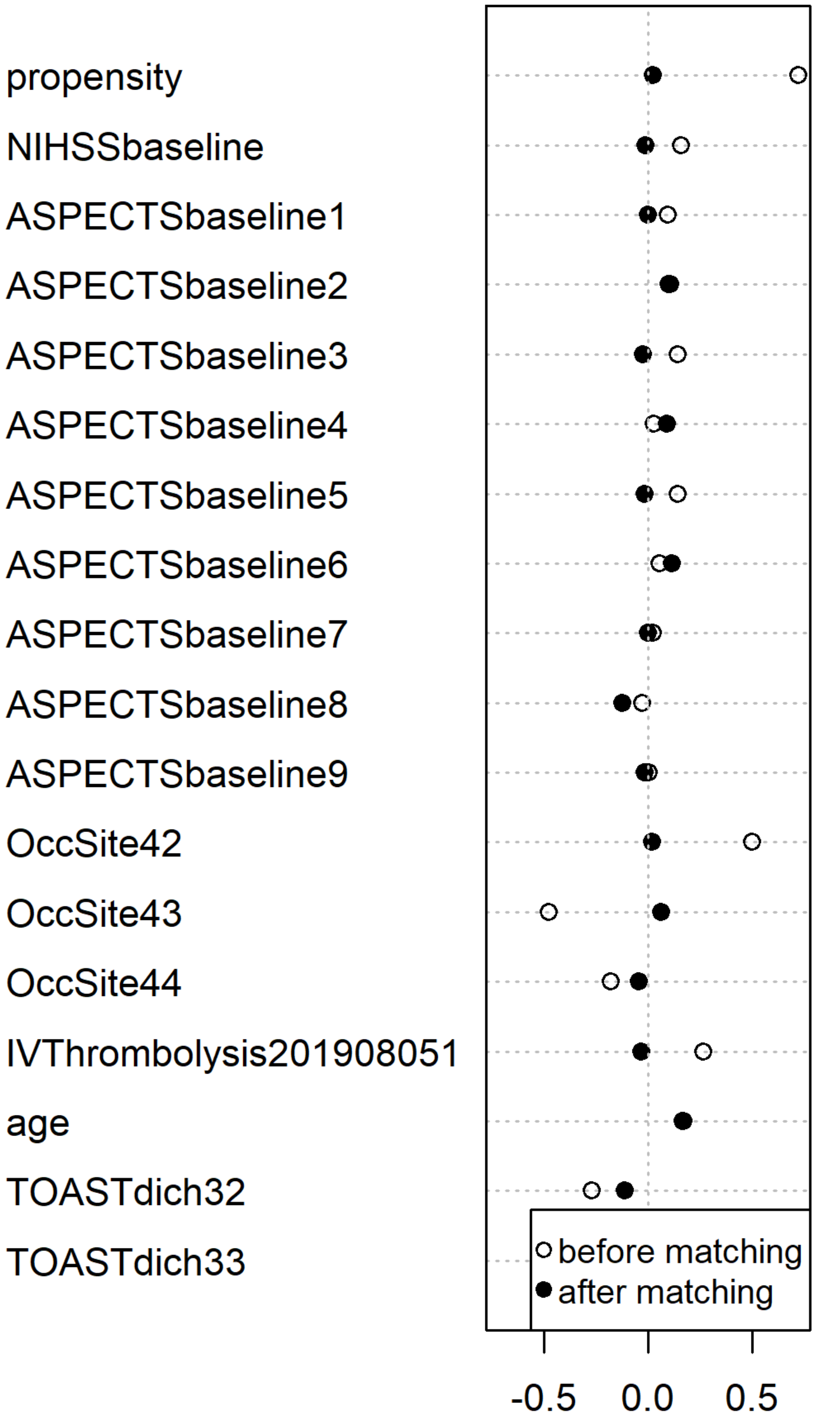

© 2021 Kong W et al. *JAMA Network Open.*

eMethods 2. Missing or Incomplete Data

Among 542 patients, six patients had missing baseline posterior circulation-Acute Stroke Prognosis Early CT Score (pc-ASPECTS), seven patients missing body temperature and three patients missing systolic blood pressure (SBP) and diastolic blood pressure (DBP). We excluded the missing essential data from our analysis, so we didn't impute for missing data.

© 2021 Kong W et al. *JAMA Network Open.*

eTable 1. Clinical Outcomes of Patients with Severe Acute Basilar Artery Occlusion by Treatment

Characteristic	Unmatched patients, No. (%)							Propensity score–matched patients, No. (%)			
	All (n=542)	EVT (n=431)	SMT (n=111)	Unadjusted OR (95% CI)	P Value	Adjusted OR (95% CI) [c]	P Value	All (n=216)	EVT (n=108)	SMT (n=108)	P Value
Primary outcomes, n/total n (%)											
90d mRS, median (IQR)	6 (4–6)	6 (4–6)	6 (6–6)	3.48 (2.08–5.83) [a]	<0.001	3.44 (2.05–5.78) [a]	<0.001	6 (5–6)	6 (5–6)	6 (6–6)	0.001 [a]
Secondary outcomes, n/total n (%)											
90d mRS											
Excellent outcomes	53 (9.8)	50 (11.6)	3 (2.7)	4.72 (1.45–15.44) [b]	0.01	3.20 (0.89–11.57)	0.08	10 (4.6)	7 (6.5)	3 (2.8)	0.20 [c]
Functional independence	77 (14.2)	74 (17.2)	3 (2.7)	7.46 (2.31–24.14) [b]	0.001	5.55 (1.59–19.38)	0.007	13 (6.0)	10 (9.3)	3 (2.8)	0.05 [c]
Favorable outcomes	98 (18.1)	93 (21.6)	5 (4.5)	5.83 (2.31–14.72) [b]	<0.001	4.52 (1.64–12.43)	0.004	18 (8.3)	13 (12.0)	5 (4.6)	0.05 [c]
90d Mortality	330 (60.9)	239 (55.5)	91 (82)	0.27 (0.16–0.46) [b]	<0.001	0.27 (0.15–0.50)	<0.001	156 (72.2)	67 (62.0)	89 (82.4)	0.001 [c]
sICH	41 (7.7)	40 (9.5)	1 (0.9)	11.55 (1.57–84.96) [b]	0.02	10.26 (1.34–78.62)	0.03	10 (4.7)	9 (8.7)	1 (0.9)	0.008 [c]
Any ICH	66 (12.2)	65 (15.1)	1 (0.9)	19.54 (2.68–142.41) [b]	0.003	17.04 (2.28–127.55)	0.006	12 (5.7)	11 (10.6)	1 (0.9)	0.002 [c]
Recanalization	350 (64.6)	341 (79.1)	9 (8.1)	42.94 (20.90–88.22) [b]	<0.001	45.60 (21.21–102.36)	<0.001	94 (43.5)	86 (79.6)	8 (7.4)	<0.001 [c]

[a] P value obtained using univariate ordinal logistic regression.
[b] The odds ratios were estimated from a binary logistic regression model.
[c] P value obtained using the Pearson test.
Abbreviations: ABAO, acute basilar artery occlusion; IQR, interquartile; OR, odds ratio; CI, confidence interval; EVT, endovascular treatment; SMT, standard medical treatment; Sich, symptomatic intracranial hemorrhage.

© 2021 Kong W et al. *JAMA Network Open.*

eTable 2. Multivariate Analysis of Estimators of Outcomes

Variables	Unadjusted OR (95% CI)	P Value	Adjusted OR[a] (95% CI)	P Value
Favorable outcome				
Age (Per 10 units)	0.91 (0.86–1.09)	0.31	0.95 (0.75–1.19)	0.63
SBP (Per 10 units)	0.93 (0.85–1.01)	0.09	0.91 (0.81–1.01)	0.07
Baseline NIHSS	0.90 (0.86–0.94)	<0.001	0.90 (0.85–0.95)	<0.001
Body temperature	0.37 (0.22–0.63)	<0.001	0.45 (0.24–0.83)	0.01
PC-CS score	1.20 (1.07–1.35)	0.002	1.09 (0.87–1.36)	0.46
pc-ASPECTS score	1.70 (1.45–2.00)	<0.001	1.69 (1.41–2.04)	<0.001
BATMAN score	1.19 (1.06–1.34)	0.002	1.13 (0.92–1.40)	0.25
Occlusion sites				
Distal BA[b]	1 [Reference]	NA	1 [Reference]	NA
Middle BA	0.39 (0.22–0.68)	0.001	0.34 (0.17–0.72)	0.004
Proximal BA	0.59 (0.30–1.16)	0.13	0.50 (0.21–1.22)	0.13
VA-V4	0.46 (0.24–0.60)	0.02	0.43 (0.18–1.03)	0.06
IVT	0.89 (0.53–1.50)	0.66	0.96 (0.52–1.77)	0.89
EVT	5.83 (2.31–14.73)	<0.001	3.80 (1.36–10.58)	0.01
Mortality				
Age (Per 10 units)	1.20 (1.03–1.39)	0.02	1.15 (0.95–1.38)	0.15
SBP (Per 10 units)	1.09 (1.02–1.17)	0.02	1.10 (1.01–1.19)	0.03
Body temperature	1.83 (1.33–2.52)	<0.001	1.53 (1.06–2.22)	0.03
Baseline NIHSS	1.13 (1.09–1.18)	<0.001	1.12 (1.08–1.17)	<0.001
PC-CS score	0.85 (0.78–0.94)	0.001	0.91 (0.77–1.08)	0.28
pc-ASPECTS score	0.73 (0.65–0.81)	<0.001	0.76 (0.67–0.85)	<0.001
BATMAN score	0.84 (0.77–0.92)	<0.001	0.90 (0.77–1.06)	0.22
IVT	0.93 (0.62–1.39)	0.72	0.79 (0.49–1.27)	0.34
EVT	0.27 (0.16–0.46)	<0.001	0.29 (0.16–0.54)	<0.001

[a] Adjusted estimates of effect were calculated using multiple regression taking the following variables into account: age, sex, EVT, SBP, smoking, Atrial fibrillation, baseline NIHSS score, PC-CS score, baseline pc-ASPECTS score, BATMAN score, stroke etiology, occlusion site, intravenous thrombolysis, and OTT.

[b] Distal basilar artery was taken as reference.

Abbreviations: ABAO, acute basilar artery occlusion, OR, odds ratio; CI, confidence interval; NIHSS, National Institutes of Health Stroke Scale; SBP, systolic blood pressure; pc-ASPECTS, posterior circulation-Alberta Stroke Program Early CT Score; PC-CS, posterior circulation collateral score; BATMAN, basilar artery on Tomography Angiography; EVT, endovascular treatment; OTT, onset-treatment time; BA, basilar artery; VA-V4, vertebral artery V4 segment.

Age OR (per 1-year increase in age)

NIHSS, PC-CS, pc-ASPECTS, BATMAN OR (per 1-point increase in NIHSS, PC-CS, pc-ASPECTS, BATMAN)

Body temperature OR (per 1-degree increase in Body temperature)

IVT OR (IVT vs no IVT)

© 2021 Kong W et al. *JAMA Network Open.*

Recanalization OR (Recanalization vs no Recanalization)

© 2021 Kong W et al. *JAMA Network Open.*

eTable 3. Comparison of Patients With Favorable vs Poor Outcomes

Characteristic	All patients, No. (%) (n=542)	Favorable outcome, No. (%) (n=98)	Poor outcome, No. (%) (n=444)	P value
Age (years), median (IQR)	65 (57–74)	64 (55–72.25)	65 (58–74)	0.32
Sex (men), n (%)	395 (72.9)	68 (69.4)	327 (73.6)	0.39
Baseline NIHSS, median (IQR)	30 (27–35)	28 (24–32)	32 (28–35)	<0.001
pc-ASPECTS baseline, median (IQR)[a]	8 (6–9)	9 (8–10)	7 (6–8)	<0.001
pc-CS score, median (IQR)	4 (3–6)	5 (4–6)	4 (2–5)	0.001
BATMAN, median (IQR)	4 (2–5)	4 (3–5.25)	3 (2–5)	0.001
Body temperature, median (IQR)[b]	36.7 (36.5–36.9)	36.5 (36.3–36.7)	36.6 (36.5–36.9)	<0.001
SBP, mean (SD)[c], mm Hg	153 (25.7)	149 (23.9)	151 (26.0)	0.08
DBP, mean (SD)[c], mm Hg	86 (15.6)	85 (15.2)	85 (15.6)	0.42
Vascular risk factor, n (%)				
Smoking current/past	179(33.0)	38(38.8)	141(31.8)	0.18
Hypertension	388(71.6)	71(72.4)	317(71.4)	0.84
Hyperlipidemia	173(31.9)	30(30.6)	143(32.2)	0.76
Diabetes mellitus	128(23.6)	16(16.3)	112(25.2)	0.06
Drinking	123(22.7)	28(28.6)	95(21.4)	0.13
Medical history, n (%)				
Atrial fibrillation	120(22.1)	26(26.5)	94(21.2)	0.25
Coronary artery disease/prior MI	103(19.0)	19(19.4)	84(18.9)	0.92
Heart failure	19(3.5)	5(5.1)	14(3.2)	0.34
Prodrome	252(46.5)	48(49)	204(45.9)	0.59
Cerebral infarction	134(24.7)	19(19.4)	115(25.9)	0.18
Intracerebral hemorrhage	10(1.8)	1(1.0)	9(2.0)	0.50
Chronic bronchitis	8(1.5)	0(0)	8(1.8)	0.18
Stroke etiology, n (%)				0.61
LAA	339(62.5)	56(57.1)	283(63.7)	
CE	143(26.4)	31(31.6)	112(25.2)	
Other causes	60(11.1)	11(11.2)	49(11.0)	
Occlusion sites, n (%)				0.003
Distal BA	188(34.7)	47(48.0)	141(31.8)	
Middle BA	175(32.3)	21(21.4)	154(34.7)	
Proximal BA	76(14.0)	13(13.3)	63(14.2)	
VA-V4	103(19.0)	17(17.3)	86(19.4)	
intravenous thrombolysis, n (%)	131(24.2)	22(22.4)	109(24.5)	0.66
OTT, median (IQR), min	311(141–402)	227.5(129.75–329)	251(141–413)	0.18
Successful recanalization, n (%)	350(64.6)	91(92.9)	259(58.3)	<0.001

[a] Data were missing for 4 patients in the EVT cohort and 2 patients in the SMT alone cohort.

[b] Data were missing for 6 patients in the EVT cohort and 1 patient in the SMT alone cohort.

© 2021 Kong W et al. *JAMA Network Open.*

[c] Data were missing for 2 patients in the EVT cohort.

Abbreviations: BAO, basilar artery occlusion; IQR, interquartile; SD, standard deviation; NIHSS, National Institutes of Health Stroke Scale; pc-ASPECTS, posterior circulation-Alberta Stroke Program Early CT Score; PC-CS, posterior circulation collateral score; BATMAN, basilar artery on Tomography Angiography; SBP, systolic blood pressure; DBP, diastolic blood pressure; MI, myocardial infarction; OTT, onset-treatment time; BA, basilar artery; VA-V4, vertebral artery V4 segment; LAA, Large artery atherosclerosis; CE, Cardioembolism.

© 2021 Kong W et al. *JAMA Network Open.*

eTable 4. Baseline Characteristics of Patients with Endovascular Therapy

Characteristic	All patients, No. (%) (n=647)	NIHSS >=21, No. (%) (n=431)	NIHSS 10~20, No. (%) (n=147)	NIHSS <10, No. (%) (n=69)	P value
Age (years), median (IQR)	64 (56–73)	65 (57–74)	63 (55–73)	64 (57–69)	0.25
Sex, n (%)					0.77
Women	164 (25.3)	111(25.8)	38 (25.9)	15 (21.7)	
Men	483 (74.7%)	320 (74.2%)	109 (74.1%)	54 (78.3%)	
Baseline NIHSS, median (IQR)[a]	27 (17–33)	31 (27–35)	15 (14–18)	6 (5–8)	<0.001
pc-ASPECTS, median (IQR)	8 (7–9)	8 (6–9)	8 (7–10)	8 (7–10)	<0.001
pc-CS score, median (IQR)	4 (3–6)	4 (3–5)	6 (4–7)	5 (4–7)	<0.001
BATMAN, median (IQR)	4 (2–6)	3 (2–5)	5 (4–6)	5 (4–6)	<0.001
SBP, mean (SD)[b], mm Hg	150 (26)	150 (26)	150 (25)	149 (24)	0.93
DBP, mean (SD)[b], mm Hg	85 (16)	85 (16)	86 (15)	84 (15)	0.10
Vascular risk factor, n (%)					
Smoking current/past	235 (36.3%)	160 (37.1%)	42 (28.6%)	33(47.8%)	0.02
Hypertension	451 (69.7%)	308 (71.5%)	100 (68.0%)	43 (62.3%)	0.27
Hyperlipidemia	214 (33.1%)	133 (30.9)	55 (37.4)	26 (37.7%)	0.24
Diabetes mellitus	149 (23.0%)	103 (23.9%)	34 (23.9%)	12 (17.4%)	0.49
Drinking	141 (21.8%)	102 (23.7%)	21 (14.3%)	18 (26.1%)	0.04
Medical history, n (%)					
Atrial fibrillation	136 (21.0%)	104 (24.1%)	21 (14.3%)	11 (15.9%)	0.02
Coronary artery disease	105 (16.2%)	87 (20.2%)	11 (7.5%)	7 (10.1%)	0.001
Heart failure [c]	22 (3.4%)	17 (3.9%)	4 (2.7%)	1 (1.4%)	0.67
Prodrome	302 (46.7%)	197 (45.7%)	68 (46.3%)	37 (53.6%)	0.47
Cerebral infarction	140 (21.6%)	101 (23.4%)	29 (19.7%)	10 (14.5%)	0.20
Intracerebral hemorrhage [c]	12 (1.9%)	8 (1.9%)	4 (2.7%)	0 (0)	0.43
Chronic bronchitis [c]	13 (2.0%)	8 (1.9%)	3 (2.0%)	2 (2.9%)	0.75
Cause of stroke, n (%)					0.19
LAA	418 (64.6%)	268 (762.2%)	101 (68.7%)	49 (71.0%)	
CE	173 (26.7%)	123 (28.5%)	32 (21.8%)	18 (26.1%)	
Other causes	56 (8.7%)	40 (9.3%)	14 (9.5%)	2 (2.9%)	
Occlusion sites, n (%)					0.08
Distal BA	222 (34.3%)	164 (38.1%)	36 (24.5%)	22 (31.9%)	
Middle BA	195 (30.1%)	117 (27.1%)	56 (38.1%)	22 (31.9%)	
Proximal BA	107 (16.5%)	70 (16.2%)	27 (18.4%)	10 (14.5%)	
VA-V4	123 (19.0%)	80 (18.6%)	28 (19.0%)	15 (21.7%)	
intravenous thrombolysis, n (%)	119 (18.4%)	82 (19.0%)	30 (20.4%)	7 (10.1%)	0.16
OTT, median (IQR), min	246 (132–390)	247 (144–401)	215 (112–341)	293 (89–420)	0.21
OTP, median (IQR), min	328 (220–493)	325 (223–495)	310 (203–475)	351 (265–618)	0.006
OTR, median (IQR), min	441 (328–626)	436 (328–623)	432 (320–613)	470 (345–749)	0.007

© 2021 Kong W et al. *JAMA Network Open.*

PTR, median (IQR), min	105 (71–151)	104 (72–146)	109 (72–169)	103 (66–166)	0.13
DTP, median (IQR), min	131 (88–200)	125 (83–180)	142 (94–215)	155 (108–280)	<0.001
DTR, median (IQR), min	247 (186–345)	234 (180–319)	265 (195–387)	300 (194–425)	<0.001
General anesthesia, n (%)	257 (39.7%)	194 (45.0%)	50 (34.0%)	13 (18.8%)	<0.001
Successful recanalization, n (%)	52 2(80.7%)	341 (79.1%)	119 (81.0%)	62 (89.9%)	0.11

[a] Data were missing for 4 patients in the EVT cohort.

[b] Data were missing for 2 patients in the EVT cohort.

[c] Fisher exact test.

Abbreviations: ABAO, acute basilar artery occlusion; IQR, interquartile; SD, standard deviation; NIHSS, National Institutes of Health Stroke Scale; pc-ASPECTS, posterior circulation-Alberta Stroke Program Early CT Score; PC-CS, posterior circulation collateral score; BATMAN, basilar artery on Tomography Angiography; SBP, systolic blood pressure; DBP, diastolic blood pressure; MI, myocardial infarction; OTT, onset-treatment time; BA, basilar artery; VA-V4, vertebral artery V4 segment; LAA, Large artery atherosclerosis; CE, Cardioembolism.

© 2021 Kong W et al. *JAMA Network Open.*

eTable 5. Multivariate Analysis of Estimators of Outcomes After Endovascular Therapy

Variables	Functional outcome		Mortality	
	Adjusted OR[a] (95% CI)	p-value	Adjusted OR[a] (95% CI)	p-value
Dichotomized baseline NIHSS	0.29 (0.19–0.46)	<0.001	2.67 (1.74–4.10)	<0.001
Age (Per 10 units)	0.88 (0.73–1.06)	0.18	1.18 (0.99–1.41)	0.06
PC-CS score (Per 1-point)	1.03 (0.87–1.22)	0.71	0.94 (0.81–1.10)	0.44
pc-ASPECTS score (Per 1-point)	1.67 (1.44–1.92)	<0.001	0.74 (0.65–0.83)	<0.001
BATMAN score (Per 1-point)	1.17 (1.00–1.38)	0.06	0.92 (0.79–1.06)	0.24
Smoking	1.40 (0.87–2.23)	0.16	0.87 (0.57–1.33)	0.51
SBP (Per 10 units)	0.92 (0.85–1.00)	0.05	1.10 (1.02–1.19)	0.01
Atrial fibrillation	0.85 (0.37–1.95)	0.69	0.84 (0.38–1.84)	0.66
Intravenous thrombolysis	0.85 (0.52–1.40)	0.53	0.91 (0.58–1.43)	0.69
Cause of stroke				
LAA[b]	1 [Reference]	NA	[1 Reference]	NA
CE	1.07 (0.48–2.38)	0.88	1.32 (0.62–2.84)	0.47
Other causes	1.16 (0.54–2.49)	0.71	0.63 (0.30–1.35)	0.24
Occlusion sites	reference	0.007	reference	0.69
Distal BA[c]				
Middle BA	0.38 (0.22–0.68)	0.001	1.17 (0.69–2.00)	0.56
Proximal BA	0.66 (0.34–1.29)	0.22	0.92 (0.48–1.77)	0.79
VA-V4	0.45 (0.23–0.87)	0.02	1.28 (0.70–2.33)	0.43
Successful recanalization	4.71 (2.41–9.21)	<0.001	0.14 (0.08–0.24)	<0.001

[a] Adjusted estimates of effect were calculated using multiple regression taking the following variables into account: age, sex, SBP, smoking, Atrial fibrillation, baseline NIHSS score, PC-CS score, baseline pc-ASPECTS score, BATMAN score, stroke etiology, occlusion site, intravenous thrombolysis, and OTT.

[b] LAA was taken as reference.

[c] Distal BA was taken as reference.

Abbreviations: OR, odds ratio; CI, confidence interval; NIHSS, National Institutes of Health Stroke Scale; SBP, systolic blood pressure; pc-ASPECTS, posterior circulation-Alberta Stroke Program Early CT Score; PC-CS, posterior circulation collateral score; BATMAN, basilar artery on Tomography Angiography; EVT, endovascular treatment; OTT, onset-treatment time; BA, basilar artery; VA, vertebral artery V4 segment; LAA, Large artery atherosclerosis; CE, Cardioembolism.

© 2021 Kong W et al. *JAMA Network Open.*

eFigure 1. Flowchart

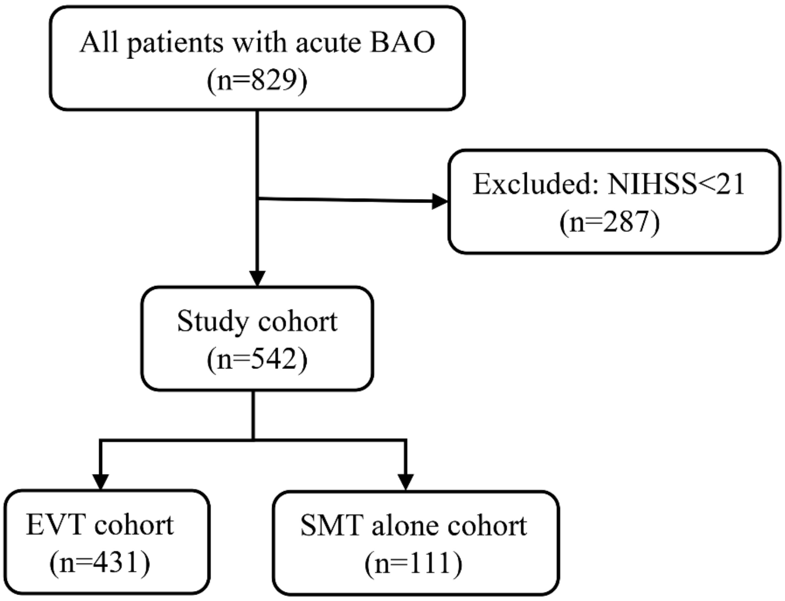

This figure shows the enrollment information of patients in the present study.

BAO, basilar artery occlusion; NIHSS, National Institutes of Health Stroke Scale; EVT, endovascular therapy; SMT, standard medical treatment.

© 2021 Kong W et al. *JAMA Network Open.*

eFigure 2. Association of Baseline National Institutes of Health Stroke Scale Score With Probability of Favorable Outcome

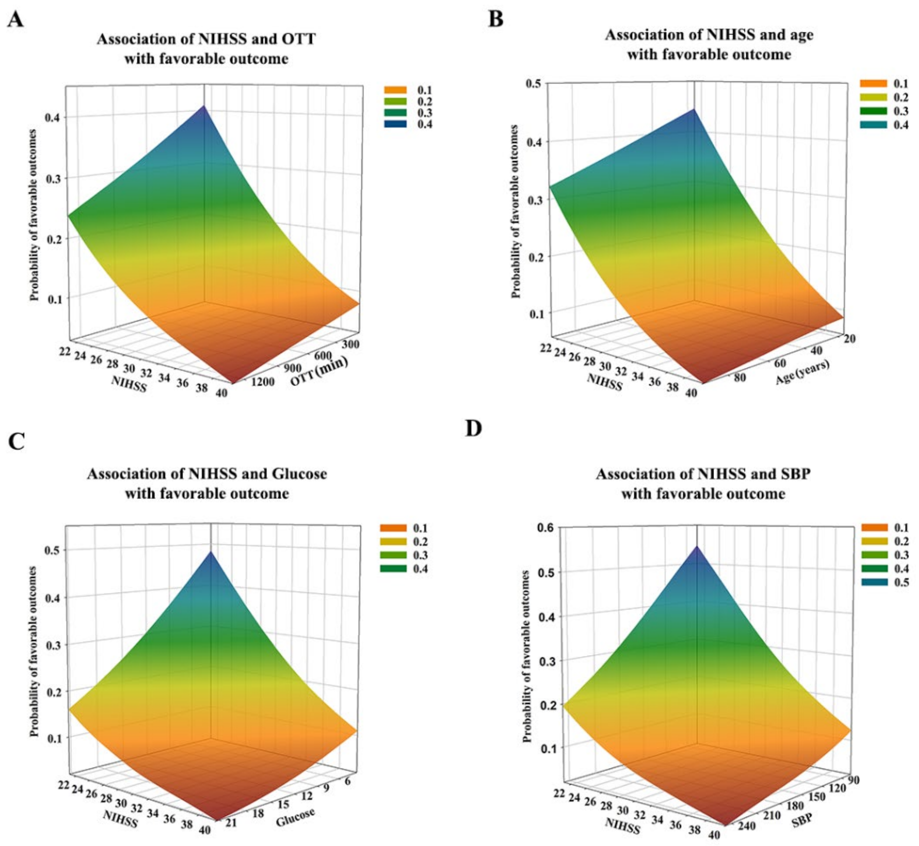

Association between baseline NIHSS and OTT **(A)**, age **(B)**, glucose **(C)** and SBP **(D)** with the probability of mortality at 90 days after EVT in the severe ABAO. The probability of favorable outcome is associated with baseline NIHSS score. There is a decrease in the likelihood of favorable outcomes with the baseline NIHSS progresses while the outcome probability is roughly stable over the onset to treatment time (OTT), age, glucose and SBP. SBP, systolic Blood Pressure.

© 2021 Kong W et al. *JAMA Network Open.*

eFigure 3. Estimated Marginal Effects of Favorable Outcome Probabilities on Onset to Puncture (OTP) Time and Puncture to Recanalization (PTR) Time

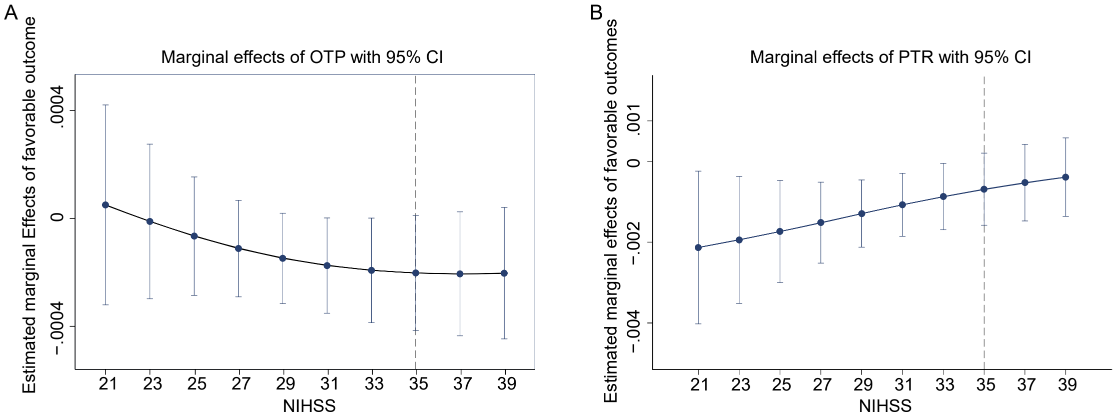

The estimated marginal effects of favorable outcome probabilities on onset to puncture (OTP) time (A) and puncture to recanalization (PTR) time (B) with increases of the baseline NIHSS in EVT group.

© 2021 Kong W et al. *JAMA Network Open.*

eFigure 4. Association of Baseline National Institutes of Health Stroke Scale Score With Probability of Clinical Outcomes With Endovascular Therapy

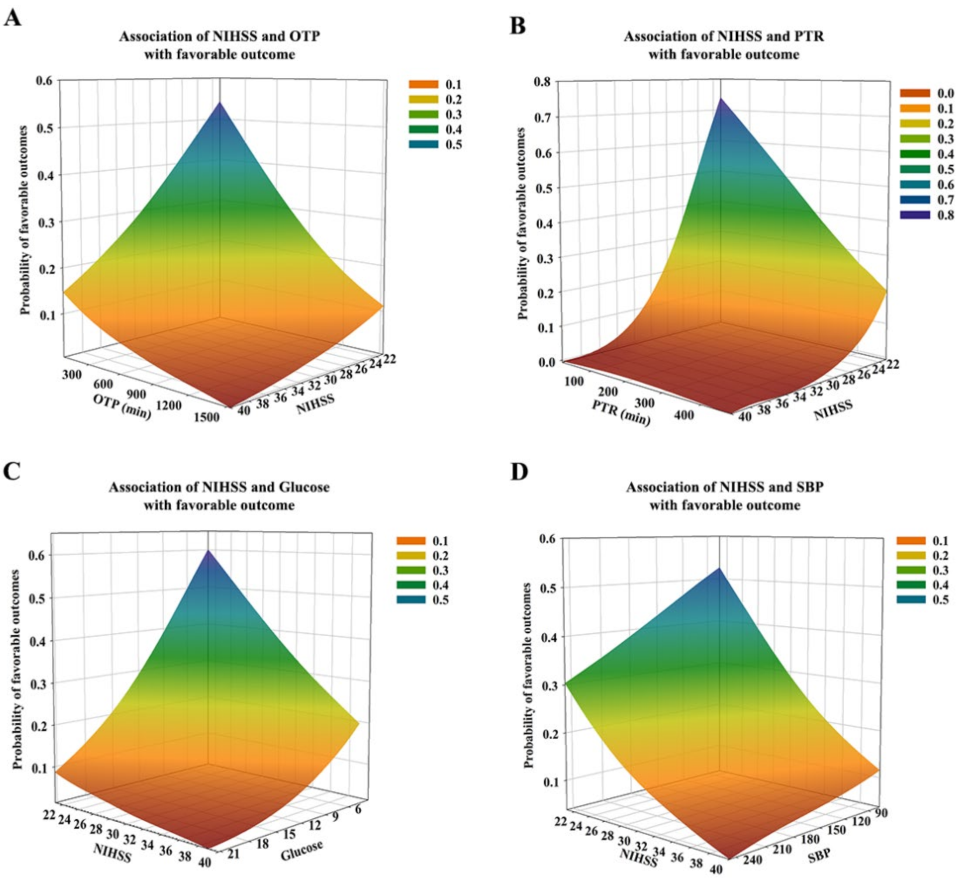

Association between baseline NIHSS and OTP **(A)**, PTR **(B)**, glucose **(C)**, and SBP **(D)** with the probability of favorable outcomes at 90 days after EVT in the severe ABAO. The probability of favorable outcome is associated with baseline NIHSS score. There is a decrease in the likelihood of a favorable outcome with increase in the baseline NIHSS score while the outcome probability is roughly stable over the onset to puncture (OTP) time, puncture to recanalization (PTR) time, glucose and systolic blood pressure (SBP).

© 2021 Kong W et al. *JAMA Network Open.*
